# Does chronic low-dose aspirin use benefit bone health? A cross-sectional study on patients with type 2 diabetes mellitus

**DOI:** 10.1186/s12902-023-01309-2

**Published:** 2023-04-07

**Authors:** Li Zhang, Xuelei Ji, Jun Chen, Yu Zhu, Zhen Wang, Zhen Ma, Yu Wu, Faguo Wu, Zhangan Zheng

**Affiliations:** 1 Department of Geriatrics, the Second People’ Hospital of Wuhu, Anhui, 230032 China; 2 Department of Endocrinology, the Second People’ Hospital of Wuhu, Anhui, 230032 China; 3 School of Basic Medical Science, and the, First Clinical Medical College, Anhui Medical University, 81# Mei Shan Road, Hefei, 230032 Anhui China; 4 Department of Trauma and Spine Surgery, the Second People’ Hospital of Wuhu, Anhui, 230032 China

**Keywords:** Nonsteroidal anti-inflammatory drugs, Bone, Aspirin, Cyclo-oxygenase, Osteoporosis

## Abstract

**Introduction:**

Numerous studies have reported the striking result that aspirin use is associated with higher bone mineral density (BMD), suggesting its potential as a population-wide osteoporosis prevention measure. Therefore, this study aimed to examine the impact of chronic low-dose aspirin use on bone remodeling biomarkers and BMD in an aging population.

**Materials and methods:**

Between September and November of 2019, clinical data regarding the medication use, serum bone remodeling biomarkers, and BMD of 567 consecutively hospitalized patients, a minimum of 50 years old with type 2 diabetes mellitus (T2DM), were collected. The cross-sectional associations between chronic low-dose aspirin use and serum concentrations of bone remodeling biomarkers and BMD were estimated separately using linear regression. Potential confounding variables were controlled for, including age, sex, and comorbidities.

**Results:**

Low-dose aspirin users had significantly lower serum bone alkaline phosphatase (BAP) concentrations than non-users (82.44 ± 28.03 U/L vs 90.71 ± 32.79 U/L, *p* = 0.025). On the other hand, low-dose aspirin users had insignificantly higher vertebral BMD (0.95 ± 0.19 vs 0.91 ± 0.21, *p* = 0.185), femoral neck BMD (0.80 ± 0.15 vs 0.78 ± 0.17, *p* = 0.309) and Ward’s triangle BMD (0.46 ± 0.14 vs 0.44 ± 0.13, *p* = 0.209), regardless of adjustment.

**Conclusions:**

This cross-sectional study demonstrated that chronic use of low-dose aspirin was associated with significantly lower serum concentrations of BAP in hospitalized patients with T2DM. The mechanism causing the insignificantly higher BMD observed in chronic aspirin users in this study and the significant increments in BMD reported in previous studies requires further clarification in other clinical trials.

## Introduction

Bone remodeling plays a vital role in renewing and sustaining the integrity of bones throughout the whole life. In specific health states, such as the menopause in women or general ageing in both sexes, the process of bone remodeling can be accelerated resulting in an imbalance between osteoclastic bone resorption and osteoblastic bone formation [1]. With age, this will lead to microstructural deterioration and decreasing bone volume, which creates a marked deficit in the structural strength of bone and increases fragility.

Aspirin, also known as acetylsalicylic acid (ASA), is one of the most commonly used medicines, with an estimated 40,000 metric tons of ASA produced per year globally [2]. Different doses of aspirin may exhibit diverse biological effects, and to date, the precise regulatory mechanisms are yet to be fully elucidated. Generally, low-dose aspirin (75–100 mg per day) inhibits the COX-1 isozyme more robustly than COX-2, and it is commonly prescribed for the prevention of cardiovascular disease (CVD) [3]. Conversely, high-dose aspirin (> 1000 mg per day) more potently inhibits COX-2 than COX-1, suppressing the conversion of prostaglandin E2 [3] and therefore it is widely taken to alleviate pain and the inflammatory response [4].

Recently, various studies have growing interests in the potential for aspirin to offer broader medical benefits, rather than simply cardiovascular protection, particularly in the prevention of age-related conditions [5], including osteoarthritis [6], neurodegenerative diseases [7, 8], age-related hearing loss [9], and age-related macular degeneration [10]. Increasingly, studies [11–22] have begun to focus on the relationship between aspirin use and bone biology and have discovered that aspirin use is highly correlated with bone remodeling and bone mineral density (BMD).

Positive results of aspirin use on BMD suggest that it may exert beneficial effects on bone health and may even prevent osteoporosis (comparably to how widely it is currently used to prevent cardiovascular events). According to the literature the exact effects of low-dose aspirin on bone remodeling in preclinical studies remains unclear, and to date no human studies have been designed to investigate the impact of low-dose aspirin on bone remodeling. Considering the high prevalence of low aspirin use amongst the elderly population, who are most susceptible to osteoporosis, investigating its potential impact upon bone remodeling and BMD is of high clinical value. Therefore, the aim of this study was to examine the precise impacts of low-dose aspirin use on bone remodeling biomarkers and BMD.

## Methodology

### Subjects

This study was designed as a single center, hospital-based, cross‐sectional study involving consecutively hospitalized patients with type 2 diabetes mellitus (T2DM) admitted to the Second People’s Hospital of Wuhu. Only patients who consented to participate in the study were included. T2DM was diagnosed according to the diagnostic criteria of the American Diabetes Association [23]. The study period spanned three months, between September and November of 2019. The primary inclusion criteria were as follows: (1) patients are aged ≥ 50 years at the time when informed consent was obtained; (2) patients have a diagnosis of T2DM. Participants were excluded if they had: (1) concurrent treatment for a malignancy; (2) clinical diagnosis of type 1 diabetes (diagnosis of diabetes and insulin use before the age of 35); (3) concurrent medication likely to influence skeletal metabolism (excluding calcium supplements of 500 mg daily or below); (4) known malabsorptive states; (5) underlying metabolic bone disease (e.g., osteomalacia); (6) calcium disorders (other than primary hyperparathyroidism); (7) chronic kidney disease. This study was approved by the Institutional Review Boards of the Second People’s Hospital of Wuhu and was conducted in accordance with the Declaration of Helsinki. All participants provided full written informed consent to the use of the collected data in all analyses.

**Fig. 1:**
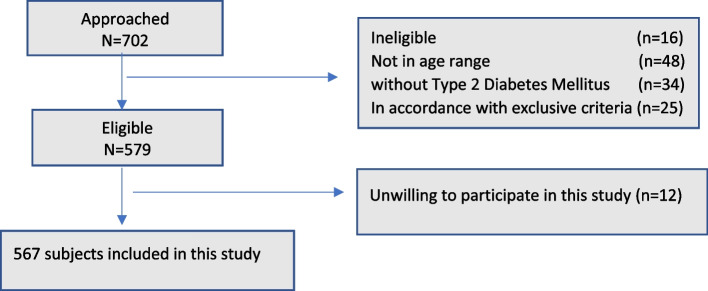
The flow of study participants that were eligible and finally included in the study

Data collectors approached 702 subjects throughout the study period. Of these, 135 were excluded from the study, thus a total of 567 patients with a median age of 69 years were enrolled in the study (Fig. 1).

### Data collection and medication use

Detailed and comprehensive assessments of each participant were carried out during the screening visit to the Second People’s Hospital of Wuhu, including the collection of data regarding their sex, date of birth, blood pressure, pulse, medical history, height (m), weight (kg), current medication and supplement use. Weight was measured on a digital scale (TanitaBWB-600), while wearing indoor clothing without shoes. Height was measured using a Harpenden stadiometer (Dyfed, UK). Body mass index (BMI) was calculated using weight (kg)/height (m^2^). Participants were asked to take all medications that they were currently using (including over-the-counter drugs and supplements) to the screening visit where study nurses recorded the pharmacy data, including drug name, dosage, date started, and duration. Chronic use of low-dose aspirin was defined as using aspirin (75-100 mg) for ≥ 1 year at baseline [24]. Patients received various anti-diabetic treatments: metformin (*n* = 284), subcutaneous insulin (*n* = 104), sulfonylureas (*n* = 53), glinides (*n* = 26), glitazones (*n* = 19), and dipeptidyl peptidase-4 inhibitors (*n* = 81). Furthermore, participants reported their medical history of brain diseases, pulmonary nodules, hepatobiliary diseases, hypertension, gastric diseases, osteoporosis and thyroid nodules.

### Laboratory methods

Venous blood samples were collected from each patient between 8:00 and 10:00 am following an overnight fast and were frozen at -20 °C after collection. Bone alkaline phosphatase (BAP, Hybritech, Liege, Belgium) is the enzyme that demonstrates osteoblastic activity and is the primary marker of bone formation [25]. The N-terminal peptide of type 1 collagen (PINP, Espoo, Finland) is also a specific product of proliferating osteoblasts and is consequently a marker of bone formation [25]. On the other hand, carboxy-terminal type I collagen crosslinks (CTX-1, Roche Diagnostics, Penzberg, Germany) resulting from the degradation of cross-linked telopeptides is the predominant marker of bone resorption [26].

### BMD and dual-energy X-ray absorptiometry methods

BMD, defined as total bone mineral content (g) divided by the area (cm^2^), was used to measure the quantity of calcium in the bone (bone density) and to determine the fracture risk [27]. Dual-energy X-ray absorptiometry (DXA) was employed to measure absolute BMD in g/cm^2^ for the lumbar spine (L1-L4, vertebral BMD), femoral neck (NCKBMD) and Ward's triangle (WRDBMD) using a Hologic QDR4500 Acclaim densitometer in all participants. The precision error of this instrument was 1%. Additionally, total fat mass (kg) was determined according to whole body DXA scans. All DXA scans were performed in the same center.

### Statistical analysis

Characteristics of participants were expressed as mean ± standard deviation (SD) or proportion, stratified by chronic use of low-dose aspirin. Continuous variables were initially evaluated by a normal distribution test; if they were not normally distributed, they were transformed into standard normal variables and assessed using independent t-tests. However, if the continuous variables failed to be converted into normally distributed variables, a nonparametric 2-Independent-Samples test was chosen to compare between these groups. Meanwhile, categorical variables were examined with the chi-square or Fisher’s exact test. Crude and adjusted mean differences in serum bone remodeling biomarkers and BMD values between chronic aspirin users and non-users alongside the corresponding 95% confidence intervals (CI) were calculated using linear regression analysis. Differences were anticipated between these two groups, which may influence bone biology and BMD. Therefore, adjustments were made for the following potential confounding factors: age, medical history of brain diseases or hypertension, use of calcium and vitamin D, statins, antihypertensive drugs, and acid-inhibitory drugs. *P* values below or equal to 0.05 were considered statistically significant. All statistical analyses were conducted using IBM SPSS Statistics, version 25.

## Results

### Characteristics of the study population

Of the 567 patients, 210 (37%) reported chronically using low-dose aspirin on a daily basis for a mean of 8.6 years (from one year to up to 32 years). Thecharacteristics of chronic low-dose aspirin users and non-users are displayed in Table 1. The mean age of recruited patients was 65.03 years (ranging from 50–94). Compared with non-users, chronic low-dose aspirin users were older (67.14 ± 9.72 vs 63.91 ± 11.16 years old, *p* = 0.011), and were more likely to have a self-reported history of brain diseases (42.9% vs 11.7%, *p* < 0.001) and hypertension (62.9% vs 41.2%, *p* < 0.001). Additionally, chronic low-dose aspirin users were less likely to take combined calcium and vitamin D (14.7% vs 24.3%, *p* = 0.037) and acid-inhibitory drugs (3.3% vs.14.6%, *p* = 0.001). However, they were more likely to take statins (82.8% vs 40.1%, *p* < 0.001), blood pressure medication (53.3% vs 30.5%, *p* < 0.001), and cardiovascular drugs (15.2% vs 6.2%, *p* = 0.008).

**Table 1:** Characteristics of the study population (567 patients) with and without chronic low-Aspirin use

Variable	Nonusers (*n* = 357)	Low-dose Aspirin users (*n* = 210)	*P* value
Age (years)	63.91 ± 11.16	67.14 ± 9.72	0.011
Male (%)	150 (42.0%)	94 (44.8%)	0.822
BMI (kg/m^2^)	24.64 ± 3.40	24.55 ± 3.86	0.867
**Medical history (%)**			
Brain diseases	42 (11.7)	90 (42.9)	0.000
Pulmonary nodules	29 (8.1)	17 (8.1)	1.000
Hepatobiliary diseases	53 (14.8)	29 (13.8)	0.872
Gastric diseases	35 (9.8)	8 (3.8)	0.057
Osteoporosis	60 (16.8)	30 (14.3)	0.645
Thyroid nodule	81 (22.7)	59 (28.1)	0.288
Hypertension	147 (41.2)	132 (62.9)	0.000
**Medication use (%)**			
Calcium plus Vit D	87 (24.3)	31 (14.7)	0.037
Statins	143 (40.1)	174 (82.8)	0.000^a^
Alendronate	8 (2.2)	9 (4.2)	0.316
Mecobalamin	48 (13.4)	45 (21.4)	0.062
Blood pressure medicine	109 (30.5)	112 (53.3)	0.000
NSAIDs	9 (2.5)	7 (3.3)	0.740
Acid-inhibitory drugs	52 (14.6)	7 (3.3)	0.001
Cardiovascular drugs	22 (6.2)	32 (15.2)	0.008

Brain disease defined as Dementia, Parkinson’s disease, Strokes/transient ischemic attack (TIA)

*BMI* Body mass index, *NSAIDs* Nonsteroidal anti-inflammatory drugs; ^a^Means significant difference

### Relationship between chronic low-dose aspirin use and serum levels of bone remodeling biomarkers

**Fig. 2:**
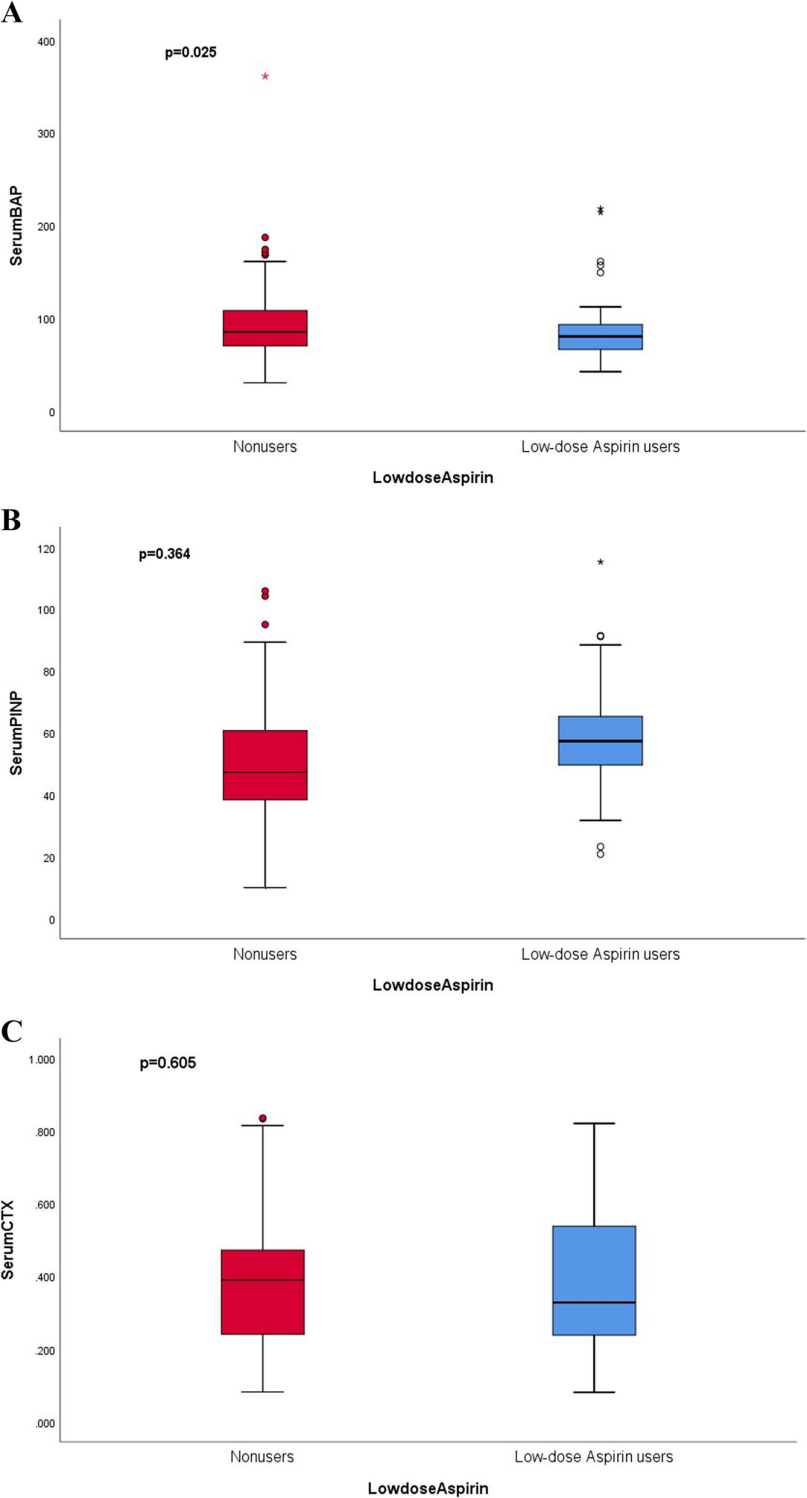
Serum bone remodelling biomarkers of chronic low-dose Aspirin users and non-users (*n* = 567). **A** BAP = Bone Alkaline Phosphatase; **B** PINP = N-terminal peptide of type 1 collagen; **C** CTX = carboxy-terminal type I collagen crosslinks

Low-dose aspirin users had significantly lower serum concentrations of BAP (82.44 ± 28.03 U/L vs 90.71 ± 32.79 U/L, *p* = 0.025) compared with non-users (as illustrated in Fig. 2A). After adjusting for age, multivariable liner regression analyses demonstrated that there were no significant differences between groups (*p* = 0.077, 95% CI -13.94–0.72). However, following full adjustment for age, brain diseases, hypertension, calcium and vitamin D intake, statin use, antihypertensive drugs, and acid-inhibitory drugs, serum BAP concentrations of low-dose aspirin users were significantly lower than non-users (*p* = 0.048, 95% CI -15.86– -0.07). This suggests that low-dose aspirin use is associated with a decreased concentration of serum BAP, the bone formation biomarker. Conversely, there were no significant differences in the serum levels of PINP and CTX-1 (low-dose aspirin users: 58.39 ± 22.26 U/L vs non-users: 49.02 ± 27.07 U/L; 0.381 ± 0.123 ng/ml vs 0.395 ± 0.115 ng/ml, respectively) (Fig. 2B and C), regardless of adjustment.

### Relationship between chronic low-dose aspirin use and BMD

BMD values at two sites of low-dose aspirin users were invariably elevated compared to those of non-users, although this difference was not statistically significant, irrespective of adjustment (Table 2). For example, low-dose aspirin users had insignificantly higher vertebral BMD (0.95 ± 0.19 vs 0.91 ± 0.21, *p* = 0.185), femoral neck BMD (0.80 ± 0.15 vs 0.78 ± 0.17, *p* = 0.309) and Ward’s triangle BMD (0.46 ± 0.14 vs 0.44 ± 0.13, *p* = 0.209) compared with non-users. Following full adjustment, there were no significant differences between aspirin users and non-users for vertebral BMD, NCKBMD and WRDBMD (Table 2). No collinearity was detected in these models.

**Table 2:** Comparisons in BMD (g/cm2) between low-dose aspirin users and non-users

Variable	Nonusers (*n* = 357)	Low-dose Aspirin users (*n* = 210)	Crude	Model 1	Model 2
vertebral BMD	0.91 ± 0.21	0.95 ± 0.19	0.185	0.727	0.998
NCKBMD	0.78 ± 0.17	0.80 ± 0.15	0.309	0.930	0.853
WRDBMD	0.44 ± 0.13	0.46 ± 0.14	0.209	0.811	0.665

Model 1: adjusted for age; Model 2: adjusted for model 1 plus Brain diseases, Hypertension, Calcium plus Vitamin D intake, statin use, antihypertensive drugs and Acid-inhibitory drugs;

*BMD* Bone mineral density, *NCKBMD* BMD of Femoral neck, *WRDBMD* BMD of Ward's triangle

## Discussion

This analysis implies that chronic low-dose aspirin use is associated with lower concentrations of serum BAP, meanwhile it is correlated with insignificantly elevated BMD, regardless of adjustment. Epidemiological studies demonstrated that T2DM is associated with an increased risk of fractures [28, 29], and suggested that T2DM should be considered as a cause of more fractures [30]. Therefore, this research only studied patients with T2DM aged over 50 years old, since this population are more susceptible to osteoporosis. This analysis, conducted in a well-documented, hospital-based clinical study, enabled the identification of and adjustment for multiple confounders.

We believe this is the first clinical study that has reported the association between low-dose aspirin use and bone remodeling biomarkers. In the literature, previous cell studies and animal experiments have explored this association but found inconsistent results. Low-dose aspirin (1–10 μM) significantly suppressed osteoblastic differentiation and decreased alkaline phosphatase (ALP) synthesis in the MG-63 cell line [12]. Nonetheless, in animal studies, aspirin-treated OVX mice (fed with 0.6 mg/ml aspirin) exhibited a significant increase in the expression of bone formation biomarkers, such as RUNX2, ALP, and osteocalcin [13]. Also, low-dose aspirin treatment (200 µg/kg) significantly raised osteocalcin levels in adult male mice [14]. This result concurred with that of higher dose of aspirin use (9.0 mg/kg/d), which significantly increased dynamic bone formation parameters (including MS/BS and Ob.S/BS) and the osteocalcin concentration in OVX rats [15]. Our study illustrated the significant, negative correlation between low-dose aspirin use and the bone formation biomarker BAP in human study, though this still requires further investigation to definitively clarify the impact on bone health.

Despite the negative correlation between low-dose aspirin use and the bone formation biomarker BAP, our study discovered that low-dose aspirin use was not associated with lower BMD, but with insignificantly higher BMD at all sites. This result was consistent with another cross-sectional analysis, The Netherland BMDs Epidemiology of Obesity (NEO) study including 6,671 residents 45 to 65 years old [22]. In this NEO study, the mean vertebral BMD and femoral BMD of low-dose aspirin users were insignificantly higher than non-users. However, the majority of other studies in the literature reported that aspirin use consistently led to significant increases in BMD [18–21]. In animal models, aspirin use at dose of 9.0 mg/kg/d [15] up to 45 mg/kg/d [17] significantly increased BMD, compared with control animals In the Health ABC cohort comprised of 2,853 adults (mean age: 73.6 years), aspirin users had significantly higher BMD across the whole body according to DXA and at both the trabecular and cortical spine according to quantitative CT [18]. In 7,786 white postmenopausal women over the age of 65, daily use of aspirin was associated with an increased BMD of the hip and spine, both prior to and following full adjustment [19]. In The Concord Health and Ageing in Men Project, which involved 1,705 men aged 70–97, aspirin use was associated with elevated spinal BMD in multivariate regression models [20]. In a partially randomized comprehensive cohort study comprised of 2,016 perimenopausal women on hormone therapy. Aspirin users presented a significant increase in spinal BMD from baseline to ten years compared to non-users [21]. It should be noted that very few of these human studies disclosed the exact dose of aspirin use and therefore it is not clear whether aspirin users in these studies are low-dose users. Considering the biological differences between low-dose and high-dose aspirin use, clinical data are lacking regarding the association between chronic low-dose aspirin use and BMD in the general population.

Based on our study, these paradoxical results regarding the impact of low-dose aspirin on bone formation and BMD might be ascribed to its multiple, yet conflicting mechanisms of action on bone metabolism. It has been reported that low-dose aspirin increases physiologically relevant nitric oxide (NO) production [1, 31], which may result in decreased osteoblast and increased osteoclast activity, in turn reducing BMD [5]. On the other hand, low-dose aspirin could increase BMD by limiting the formation of osteoclasts through inhibiting the NFκB pathway and stimulating the formation of osteoblasts by blocking apoptosis of its progenitor stem cells [32]. Thereby, in theory, aspirin can have both positive or negative effects on bone metabolism and consequently BMD. However, considering the diverse and complex biological effects of aspirin at different doses, the paradoxical results from this cross-sectional analysis must be interpreted with great caution.

Our analysis had several strengths and limitations. This study was especially designed to investigate the association between chronic low-dose aspirin use and bone remodeling biomarkers alongside BMD in a specific population group (patients with T2DM from a single center, over 50 years old). Furthermore, low-dose aspirin use was confirmed to be present for a minimum of one year to up to 32 years in the study participants, suggesting relative stability of exposure. However, the study population was comprised of a limited number of patients with T2DM and the findings may not translate into younger, healthy individuals. In this study actually all the results were statistically not significant, except serum BAP. Therefore, we should not exclude the possibility that this BAP result arose by chance and that all of the results in this study were negative. In addition, it has been reported that different anti-diabetic drugs may exert conflicting effects on bone health [33], but in this study further analyses attempting to eliminate this influencing factor were not conducted. Moreover, our study was unable to explore the impact of aspirin use on alternative bone resorption biomarkers, for example cathepsin K and TRAP (tartrate-resistant acid phosphatase), and most vitally, on fracture risks.

## Conclusion

This cross-sectional study demonstrated that chronic use of low-dose aspirin was associated with significantly lower serum concentrations of BAP in hospital-based patients with T2DM. The mechanism underpinning the insignificantly elevated BMD observed in chronic aspirin users discovered in this study in addition to the significant increase in BMD reported in previous studies, requires further clarification in future clinical trials.

## Data Availability

The datasets used or analysed during the current study are available from the corresponding author on reasonable request.
